# Persistent SARS-CoV-2 infection in asymptomatic young adults

**DOI:** 10.1038/s41392-022-00931-1

**Published:** 2022-03-09

**Authors:** Mai-Juan Ma, Shao-Fu Qiu, Xiao-Ming Cui, Ming Ni, Hong-Jie Liu, Run-Ze Ye, Lin Yao, Hong-Bo Liu, Wu-Chun Cao, Hong-Bin Song

**Affiliations:** 1 State Key Laboratory of Pathogen and Biosecurity, Beijing Institute of Microbiology and Epidemiology, Beijing, China; 2 Center for Disease Control and Prevention, Chinese People’s Liberation Army, Beijing, China; 3 Beijing Institute of Radiation Medicine, Beijing, China; 4 School of Public Health, Shandong University, Jinan, China

**Keywords:** Infectious diseases, Infectious diseases

**Dear Editor**,

While most of the patients infected with severe acute respiratory syndrome coronavirus 2 (SARS-CoV-2) cleared the virus within a few weeks of infection, some people have a persistent infection or persistent shedding viral RNA in the long term. The persistent detection of viral RNA in clinical specimens is unlikely to reflect either relapse or reinfection in most cases because the replication-competent virus is generally not recoverable after ten days following symptom onset in mild to moderate cases of COVID-19^1^ and after 20 days in severe or immunocompromised cases.^2^ However, the evidence from several studies revealed the shedding of infectious virus up to 70–105 days after the first positive detection in immunocompromised patients, and viruses sampled at different time points showed different dominant genetic mutations.^3–6^ Moreover, persistent SARS-CoV-2 infection within immunocompromised individuals could accumulate more mutations during convalescent plasma therapy with evidence of reduced susceptibility to neutralizing antibodies.^6^ However, the temporal dynamics of SARS-CoV-2 infectivity and the evolution of SARS-CoV-2 mutational profiles over prolonged infection periods in non-immunocompromised individuals with asymptomatic infection remains limited.

Between August and October 2020, 30 healthy and young adults who traveled overseas and returned to China were identified SARS-CoV-2 infection by real-time reverse transcriptase-polymerase chain reaction (RT-PCR) (Supplementary Table 1 and Supplementary methods). They showed no clinical symptoms during quarantine and treatment at a local hospital. We longitudinal collected nasopharyngeal swabs (NS) from these asymptomatic cases by an interval of 1–3 days to characterize viral shedding over time. We found that six of them had a prolonged (referred to prolonged cases 1–6, PCs 1–6) SARS-CoV-2 viral RNA shedding, with a median day of 106 (interquartile range [IQR] 100–110) for nucleocapsid gene since the first positive RT-PCR detection and the shedding of sgRNA up to 50 days for PCs 1–3 (Fig. 1a). The remaining 24 asymptomatic cases displayed a short duration of viral RNA shedding (referred to non-prolonged cases 7–30, NPCs 7–30), with a median day of 7 (IQR 3.3–22).

**Fig. 1:**
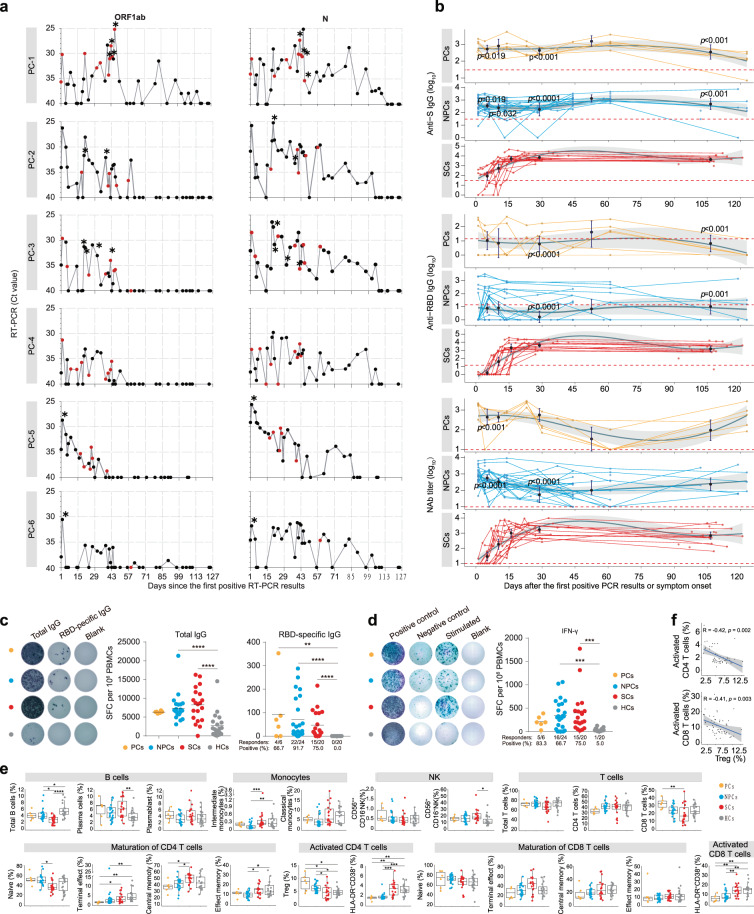
Dynamic of viral RNA shedding and antibody and cellular response in prolonged asymptomatic SARS-CoV-2 infection in young adults. **a** Dynamic of the viral load in the nasopharyngeal swab samples of six prolonged asymptomatic cases (PCs) is indicated by the cycle threshold (Ct) values for SARS-CoV2 open reading frame 1ab (OFR1ab) and nucleocapsid protein (N) of real-time reverse transcriptase-polymerase chain reaction (RT-PCR). Dots indicate individual nasopharyngeal swab samples collected at the indicated days after the first positive RT-PCR detection, and the whole viral genome successfully sequenced are indicated with red color. The asterisk indicates the positive detection of subgenomic RNA of SARS-CoV-2 in nasopharyngeal swab samples. **b** Dynamic of IgG levels against spike (S) and receptor-binding domain (RBD) of SARS-CoV-2 and pseudovirus neutralizing antibody (NAb) titer in serum from 6 PCs, 24 non-prolonged asymptomatic cases (NPCs), and 20 symptomatic cases (SCs). Dots represent individual serum samples collected at the indicated times, and the lines connect the samples from the same cases. A non-parametric loess function is shown as the blue line, with the gray-shaded representing the 95% confidence interval (CI). The geometric mean endpoint titers (purple line) and 95%CI are shown for the IgG and NAb at time points of serum sampling. **c**, **d** The total and SARS-CoV RBD-specific IgG^+^ memory B cell (**c**) and IFN-γ-producing T cell (**d**) response in PCs, NPCs, and SCs, and health controls (HCs). The left panel of **c**, **d** are representative ELISpot from each type of subject, with blank as negative control and anti-CD3 as positive controls. The right panel of **c**, **d** are cell counting in subjects. Data in the graph represented as spot-forming cells (SFC) per 1 × 10^6^ PBMCs. Each point on the dot plot represents an individual subject. The bold black line represents the mean. **e** Cell surface immune phenotyping of B, NK, DC, and T cells by flow cytometry in PCs, NPCs, SCs, and HCs. Each dot represents the percentage of cellular responses for an individual. Boxplots indicate median, interquartile range, and 95%CI. **f** Correlations between Treg and activated CD4^+^ and CD8^+^ T cell. *n* = 6, 24, 20, and 20 for PCs, NPCs, SCs, and HCs, respectively. Two-tailed Kruskal–Wallis test with false discovery rate method was used for multiple comparisons in **b**–**e**. Statistical comparisons were performed using the two-sided nonparametric Spearman correlation for **f**. *p < 0.05, **p < 0.01, ***p < 0.001, ****p < 0.0001. No asterisk indicates not significant

To explore the genetic characteristic of SARS-CoV-2 from the cases, RT-PCR positive original specimens were sequenced directly using the next-generation sequencing (Supplementary methods), and 40 high-quality whole SARS-CoV-2 genomes from NS specimens of 6 PCs and 29 from NS specimens of 18 NPCs were obtained and deposited in GenBank (accession numbers SRR13948608-SRR13948676). These 69 SARS-CoV-2 genomes belong to two lineages B.1 (13 of 69, 18.8%) and B.4 (56 of 69, 81.2%), which are relatively phylogenetic distinct in the SARS-CoV-2 global phylogeny (Supplementary Fig. 1a). The majority of cases were infected with the B.4 viruses, whereas the PC-1, NPC-7, and NPC-14 were infected with B.1 viruses. The phylogenetic relationships of the longitudinal B.4 viruses were not consistent on sampling days (Supplementary Fig. 1b–f), which indicated the diversity of the viral population and possible randomness in RNA shedding. Single nucleotide variations (SNVs) analysis showed that the B.1 viruses were characterized by the D614G mutation (A23403G) and A570D mutation (C23271A) for the B.4 viruses at spike protein, which was predicted as function conservative (Supplementary Fig. 2a, b). We observed a significantly lower cycle threshold (C_t_) value of the B.1 viruses than the B.4 viruses (Supplementary Fig. 2c, *p* = 0.00204), consistent with the previous study.^7^ While rare SNVs were observed for B.1 viruses, a high mutational load in the B.4 viruses enriched nonsynonymous SNVs in spike gene compared to the open reading frame (ORF) 1a and ORF1b genes (Supplementary Table 2).

To further evaluate the difference of antibody response between PCs and NPCs after the first positive RT-PCR detection, we measured spike (S) and receptor-binding domain (RBD) binding IgG antibodies and pseudovirus neutralizing antibodies (NAb) (Supplementary methods). We found that both PCs and NPCs displayed low endpoint titer (<1000) of S- and RBD-IgG antibodies and no obvious changes of geometric mean endpoint titer throughout infection over time (Fig. 1b). For comparison, serum samples with similar time points (≤7, ≤14, 15–21, and 28–42 days and 2–3 months post-symptom onset [PSO]) from 20 symptomatic cases (referred to SCs) were included (Supplementary Table 1). We found that both PCs and NPCs had significantly lower geometric mean endpoint titer of anti-S and anti-RBD IgG antibodies than SCs (Fig. 1b). A pattern of NAb that closely resembled anti-S and anti-RBD IgG responses was observed, but no significant difference was observed with SCs three months PSO (Fig. 1b). We observed a significant correlation between NAb and binding IgG antibodies (Supplementary Fig. 3). As expected, we observed a minimal reactivity of anti-S or anti-RBD IgG antibodies in the serum of healthy controls (HCs), but no serum samples from HCs tested positive for NAb (Supplementary Fig. 4). These findings indicate that asymptomatic cases induced a relatively low antibody response related to SCs.

We next measured the SARS-CoV-2-specific memory B cells secreting IgG and T cells secreting IFN-γ using an enzyme-linked immunospot (ELISpot) assay (Supplementary methods). The numbers of RBD-specific IgG^+^ memory B cells were significantly higher among cases than HCs (Fig. 1c). However, no significant differences were observed among case groups (Fig. 1c). RBD-specific IgG^+^ memory B cells were detected in 4 (66.7%) of 6 PCs two months after the first RT-PCR positive detection, 22 (91.7%) of 24 NPCs, and 15 (75%) of 20 SCs three months after infection (Fig. 1c). While we observed more specific T cells secreting IFN-γ in NPCs and SCs than HCs, 6 PCs showed a similar mean number of T cells secreting IFN-γ with HCs (Fig. 1d). Regarding the responder of subjects to SARS-CoV-2, we observed T cell response detectable in 83.3% (5/6), 66.7% (16/24), and 75% (15/20) of the six PCs, 24 NPCs, and 20 SCs, respectively (Fig. 1d). While there are significant correlations between anti-S and -RBD IgG antibodies and the numbers of RBD-specific IgG^+^ memory B cells, an absence correlation between the numbers of T cells secreting IFN-γ and antibody levels (Supplementary Fig. 5a, b). These results indicate that most PCs and NPCs developed and maintained specific B and T cells two months after the first RT-PCR positive detection.

We further assessed immune status in PCs and NPCs two months after first positive RT-PCR detection by immunophenotyping B cells, monocytes, NK cells, and T cells using flow cytometry (Supplementary methods). We observed a decreased frequency of B cells in both NPCs and SCs than HCs, but NPCs showed a higher frequency than SCs, of which SCs had more plasma cells than HCs (Fig. 1e). The CD14^+^CD16^+^ proinflammatory intermediate monocytes level was significantly decreased in NPCs compared to SCs and HCs (Fig. 1e). A significantly higher frequency of CD56^+^CD16^+^ NK cells in SCs than HCs was observed (Fig. 1e). While the composition of overall T cells, CD4^+^, and CD8^+^ T cells were similar between all groups (Fig. 1e), there was a marked increase of Tregs in PCs and NPCs compared to SCs and HCs (Fig. 1e). On the contrary, both PCs and NPCs have significantly reduced HLA-DR^+^CD38^+^ CD4 and CD8 T cells compared with SCs and HCs and lower frequency of terminal effect and/or central memory CD4 T cells (Fig. 1e). In addition, we observed a significant negative correlation between the percentage of CD4 Tregs and HLA-DR^+^CD38^+^ CD4^+^ (*r* = −0.41, *p* = 0.002) and CD8^+^ T cells (*r* = −0.41, *p* = 0.003) (Fig. 1f). These findings indicated a dysregulation of Treg and activated CD4^+^ and activated CD8^+^ T cells even recovered from the disease.

In summary, despite a small cohort sample size of PCs, failure isolation of the virus, and cross-section assessment of immune status in asymptomatic cases two months after first positive RT-PCR detection, our data indicate that certain PCs shed infectious viruses in the long term. Moreover, our data suggest that PCs had developed relatively low antibody response but considerable memory B and T cells and upregulation of CD4 Tregs two months after infection. Further studies are needed to assess the kinetics of immune response among PCs and NPCs and the duration of infectivity in PCs, which is necessary for improved patient management and public health benefit.

## Supplementary information

Supplemental material
