# Supplementary material for: Persistent SARS-CoV-2 infection in asymptomatic young adults
Source: Signal Transduct Target Ther. 2022 Mar 9;7:77. doi: 10.1038/s41392-022-00931-1 (PMC8905556; doi:10.1038/s41392-022-00931-1)
Supplement: Supplementary file 1 — Supplemental material [file 41392_2022_931_MOESM1_ESM.docx]

Supplementary Materials for

**Persistent SARS-CoV-2 infection in asymptomatic young adults with impaired immune responses**

Mai-Juan Ma^1^, Shao-Fu Qiu^2^, Xiao-Ming Cui^1^, Ming Ni^3^, Hong-Jie Liu^3^, Run-Ze Ye^1,4^, Lin Yao^1^, Hong-Bo Liu^2^, Wu-Chun Cao^1^**^🖂^**, Hong-Bin Song^2^**^🖂^**

**Affiliations**

^1^State Key Laboratory of Pathogen and Biosecurity, Beijing Institute of Microbiology and Epidemiology, Beijing, China

^2^Center for Disease Control and Prevention, Chinese People’s Liberation Army, Beijing, China

^3^Beijing Institute of Radiation Medicine, Beijing, China

^4^School of Public Health, Shandong University, Jinan, China

These authors contributed equally: Mai-Juan Ma, Shao-Fu Qiu, and Xiao-Ming Cui

Correspondence: [mjma@163.com](mailto:mjma@163.com) (M.J.M); [caowuchun@126.com](mailto:caowuchun@126.com) (W.C.C); [hongbinsong@263.net](mailto:hongbinsong@263.net) (H.B.S)

**This PDF file includes:**

Supplementary methods

Figures. S1 to S6

Tables S1-S4

**Supplementary methods**

**Cases and healthy controls**

Between August and October 2020, asymptomatic cases with laboratory-confirmed infection of SARS-CoV-2 by positive reverse transcription-polymerase chain reaction (RT-PCR) in China were invited to participate in this study. A total of 30 asymptomatic cases were enrolled. After the enrollment, their nasopharyngeal swabs were collected with a time interval of 1-3 days after the first positive RT-PCR results. Their blood samples for serum isolation were collected on day 1 (defined as the first positive RT-PCR results) 4, 7, 15, 60, 120 after the first positive RT-PCR results. We additional collected a 5 ml blood sample approximately 60 days after the first positive RT-PCR results for PMBC isolation, and the PBMC samples were used to detect of IFN-γ-producing T cells and IgG-secreting B cells and cell immunophenotyping. For comparison, serial serum with similar timepoints with asymptomatic cases collected from 20 symptomatic cases (≤7, ≤14, ≤21, and 28 days post-symptom onset as well as 3–4 post-symptom onset) with mild-moderate illness was used. The PBMCs collected 3 months after symptom onset of these 20 symptomatic COVID-19 cases were also included. In addition, serum and PBMCs from 20 sex- and age-matched normal healthy individuals whose samples were collected in 2018 were used as healthy controls. The demographic and clinical characteristics of the cases, including baseline demographic data, date of symptoms onset, presenting symptoms including fever, cough, sputum production and sore throat, past medical, and hospitalization, were collected at enrollment. All cases provided written informed consent. The study was conducted following the Declaration of Helsinki, and the Institutional Review Board of the Academy of Military Medical Sciences approved the study protocol (IRB number: AF/SC-08/02.60).

**Case and disease severity definition**

A laboratory-confirmed patient of COVID-19 was defined as an individual positive for SARS-CoV-2 by RT-PCR of nasopharyngeal swabs. A symptomatic patient was defined as an individual with laboratory-confirmed COVID-19 with symptoms such as fever, cough, sore throat, sputum, and so on. Asymptomatic infection was defined as an individual who had a positive SARS-CoV-2 by RT-PCR without any relevant clinical symptoms. According to the diagnostic and treatment guideline for SARS-CoV-2 issued by the Chinese National Health Committee (Version 8), mild illness was defined if the clinical symptoms were mild but without radiological signs of pneumonia; moderate illness was defined according to the following criteria: (i) fever and respiratory symptoms; (ii) radiological signs of pneumonia; severe illness was defined if satisfying at least one of the following items: (i) breathing rate ≥30/min; (ii) pulse oximeter oxygen saturation (SpO_2_) ≤93% at rest; (iii) ratio of the partial pressure of arterial oxygen (PaO_2_) to a fraction of inspired oxygen (FiO_2_) ≤300 mm/Hg (1 mm/Hg=0.133 kPa).

**Nasopharyngeal swab sample RNA extraction and RT-PCR**

All collected nasopharyngeal swabs of patient samples were stored at −80°C before being transported using the cold chain to a biosafety level 2 enhanced laboratory to perform molecular detection genome RNA (gRNA) and subgenomic RNA (sgRNA) of SARS-CoV-2. RNA was extracted from clinical samples using the viral RNA mini kit (52904, Qiagen, Hilden Germany) following the manufacturer´s instructions. Real-time reverse transcriptase-polymerase chain reaction (rRT-PCR) for SARS-CoV-2 gRNA was performed using an rRT-PCR Test Kit (20203400065, Shanghai BioGerm Medical Biotechnology Co., Ltd) targeting the Open reading frame 1ab (ORF1ab) and nucleoprotein (N genes) that are specific to SARS-CoV-2 was used for molecular testing for SARS-CoV-2. A cycle threshold (C_t_) value less than 40 was considered positive for SARS-CoV-2 RNA, and a Ct value of 40 or more was defined as a negative test. The rRT-PCR for sgRNA of SARS-CoV-2 was performed and the rRT-PCR positive for sgRNA were further vilified Sanger sequencing as previously described^1^. The RT-PCR reaction were conducted with by a one-step RT-PCT kit (Cat. No. 057A, TaKaRa, Dalian, China), forward primer in the 5’ leader region and gene-specific probes and reverse primers (sgLeadSARS-CoV-2 forward primer: 5’- CGATCTCTTGTAGATCTGTTCTC-3’; E gene-probe: 5’-FAM-ACACTAGCCATCCTTACTGCGCTTCG-ZEN-IBHQ-3′, E gene-reverse primer: 5’- ATATTGCAGCAGTACGCACACA-3’).

**SARS**-**CoV**-**2 whole viral sequencing, assembly, and phylogenetic analysis**

The longitudinal NS samples with a positive detection for both N and OFR1ab genes were subjected to next-generation sequencing. The samples were sequenced directly from the original specimens. According to the manufacturer's recommendations, viral RNA for nasopharyngeal swab samples was extracted using QIAamp Viral RNA Mini Kit (52904, QIAGEN, Heiden, Germany) A total amount of 1μg total RNA per sample was used as input material for SuperScript IV Reverse Transcriptase Kit (ThermoFisher, Foster City, CA, US) by choosing a random primer. The cDNA was used as the template for the consequence experiment. Sequencing libraries were generated using SARS-CoV-2 Multip-Seq Panel (BGI PathoGenesis Pharmaceutical Technology, BGI-Shenzhen, Shenzhen, China) ) following the manufacturer's recommendations. Unique Dual Barcode was added to each sample. This panel contains 252 amplicons/primer pairs and high genome coverage of 99.83%. Products were purified using AMPure XP beads (Beckman, Germany). The corresponding adapter oligonucleotides were joined by the second round of PCR reaction. Products were purified using AMPure XP beads (Beckman, Germany). The Qubit ® 4.0 (ThermoFisher, Foster City, CA, US) was used to determine the concentration of the library; Qsep-100 (Hangzhou Houze Biotechnology Co., Ltd.) system was used to determine the length of library fragments. The qualified library was pooled at 3nmol each. After circularization and generating DNBs, the resulting libraries were sequenced with MGI High-throughput Sequencing Set (PE150) on MGISEQ-2000/T7 platforms (MGI, Shenzhen, China). Raw reads were filtered to remove adaptors and low-quality and ambiguous bases using fastp (v0.20.1) ^2^.

A reference-based assembly of the NGS sequencing reads was conducted according to following pipeline. Briefly, raw NGS reads were firstly trimmed by using Trimmomatic V 0.39^3^ with the parameter of “HEADCROP:30” for forward reads and “CROP:120” for the reversed reads. Then the reads was aligned to the genome of SARS-CoV-2 Wuhan-hu-1 (GenBank accession MN908947.3, GISAID accession EPI_ISL_402125) by using BWA v0.7.17^4^. Based on the sam file, we filter out the reads that the match number was >80 bp. The selected reads were then aligned to the genome of SARS-CoV-2 Wuhan-hu-1^5^ .The sam file was sorted by ‘sort’ program of SAMtools v1.10^6^. Mpileup files were generated by using the ‘mpileup’ program of SAMtools v1.10 with parameters of “-A -d 10000 -B -Q 0”. The single nucleotide variations (SNVs) and their mutated allele frequencies (MuAFs) were identified as described in ref. The bioinformatics workflow for SNV calling is available at <http://github.com/generality/iSNV-calling>, which uses mpileup files as input. A Q20 quality filtering was first conducted for the sequencing bases. The MuAFs of SNVS were obtained for sites with a >= 100-hold depth and >= 5 support reads for the substitutions. Consensus sequences were then generated by using homemade script based on the single nucleotide variations whose MuAF >0.5.

Mutations of small insertion and deletion (Indels) were identified by using VarScan2 v2.4.4^7^, which uses mpileup files as input. The parameters for Indels calling were as following: “--min-coverage 100 --min-reads2 5 --min-var-freq 0.05”. At the same time, we also used Pindel(0.2.5b9)^8^ to identify indels with at least 5 reads support the mutation. The visualization tools IGV v2.4.17^9^ was used for manual examination and filtering Indels that were identified by both software. Both the SNVs and small Indels were annotated by using SnpEff v4.3t ^10^ with a reference SARS-CoV-2 Wuhan-hu-1 with default parameters.

For the samples that the sequencing coverage was more than 27000 bp, Pangolin software (v 2.1.3)^11^ was used to assign the lineage of the assembled genome on the web (<https://pangolin.cog-uk.io/>). For the other samples that the sequencing coverage was less than 25000 bp, the lineage was assigned according to the mutations specific to the two lineages. The mutations specific to the lineage B.1 are C241T, G376T, C1059T, C1917T, C3037T, C13721T, C14408T, A15370G, G17944T, A23403G, G25563T, T28889C. The mutations specific to the lineage B.4 are G1172T, G1397A, G8653T, G11083T, C23271T, T28688C, G29742T. As long as there are at least 2 mutations specific to a certain lineage in a sample and the number of mutations specific to another lineage is <= 1, then the sample was assigned to this lineage.

Multiple alignments of genome sequences were performed by using MAFFT v7.458 ^12^ and manually inspected by using MEGA v10.1.8^13^. Given the bias of genome coverage of public genome and sequences in this study, part of the 5' and 3' untranslated region was removed, and the aligned genome length was 29706 nucleotides. We explored the phylogenetic structure with the maximum likelihood (ML) method. ML Phylogenies of the global tree were inferred by using IQ-Tree2 (v 2.0.3) ^14^ with the best-fitting substitution model parameters (GTR+F+R2) estimated by Model Finder and 1000 rapid bootstrapping replicates. The genomes in this study were aligned by using MAFFT v7.458, and 28bp of the 5' and 170bp of 3' untranslated region was manually removed by using MEGA v10.1.8. Then the Phylogenetic analyses were performed by using RAxML v8.2.12 ^15^ with 1000 bootstrap replicates and employing the GTRGAMMA+I model. The generated phylogenetic trees were visualized and annotated with the iTOL (<http://itol2.embl.de/>) ^16^. The public genome datasets we used were downloaded from the GISAID database (https://www.gisaid.org/), and part of the downloaded public genome we referenced article^17^ (102 in total). We first filtered the downloaded public genome data and set the filter condition that a genome with a length of less than 29300bp and containing N bases. We selected the representative genome of each continent for the construction of the evolutionary tree.

**Serum and PMBC isolation**

Venous blood was collected from each participant to separate serum or isolate peripheral blood mononuclear cells (PBMCs). Sera were separated by centrifugation at 2000 rpm for 10 minutes, aliquoted into three cryovials, and preserved at -80°C until testing. PBMCs were isolated by density gradient centrifugation with Lymphoprep in SepMate tubes (Stemcell Technologies) according to the manufactory’s instruction. Briefly, the blood was placed on top of Lymphoprep in SepMate tubes and centrifuged at 1200 × *g* for 10 min. PBMCs from the top layer were harvested and washed twice with PBS at 400 × g for 10 min. Isolated PBMCs were frozen in cell recovery Media containing 10% DMSO (GIBCO), supplemented with 90% heat-inactivated fetal bovine serum, and stored liquid nitrogen before assays analyses.

**ELISA analysis of serum IgG antibody to RBD and Spike trimer**

To further quantify the serum IgG antibody response to RBD and S protein of SARS-CoV-2 ^18^, the recombinant RBD and S trimer derived from SARS-CoV-2 (Sino Biological, Beijing) were coated onto flat-bottom 96-well plates overnight at 4°C with a final concentration of 1μg/ml. Plates were washed with PBS-T (PBS with 0.05% Tween 20) and blocked with blocking buffer (5% skim milk and 2% BSA in PBS) for 1h at room temperature. Duplicate 3-fold 8-point serial dilutions (starting at 1:100) of heat-inactivated serum samples diluted in 1% milk in PBS-T were added to the wells and incubated at 37°C for 1h. Wells were then incubated with the secondary anti-human IgG antibody labeled with HRP (ZSGB-BIO, Beijing) and TMB substrate (Kinghawk, Beijing). The optical density (OD) was measured by a spectrophotometer at 450nm and 630nm. Endpoint antibody titers were calculated as the reciprocal serum dilution giving signal three times that of the healthy controls using a serum titration starting at 1:100 and using a 3-fold dilution series by a fitted curve (4 parameter log regression).

**SARS-CoV-2 pseudovirus neutralization assay**

SARS-CoV-2 pseudovirus was generated by co-transfection of human immunodeficiency virus backbones expressing firefly luciferase (pNL43R-E-luciferase) and pcDNA3.1 (Invitrogen) expression vectors encoding the full-length Spike proteins of SARS-CoV-2 into 293T cells (ATCC)^18^. Viral supernatants were collected 48 hrs. Later. Viral titers were measured as luciferase activity in relative light units (GloMax 96 Microplate Luminometer, Promega Biosciences). For neutralization assay, serial dilutions of patient serum samples were performed duplicated, followed by incubating pseudovirus. Plates were incubated at 37 °C for 1 h followed by the addition of Huh7/ACE2 cells (approximately 1.5 × 10^4^ per well). Wells containing cells and pseudovirus (without sample) or cells alone acted as positive and negative infection controls, respectively. Half-maximal inhibitory concentrations (IC_50_) of the serum samples were determined by luciferase activity 48 hrs after exposure to the virus-serum mixture with a three-parameter non-linear regression inhibitor curve in GraphPad Prism 8.4.1 (GraphPad Software). Titers were determined as the serum dilution that inhibited 50% virus infection (ID50).

**ELISpot assays for measurement of IgG-secreting B cells and IFN-γ-secreting T cells**

To assess B cells secreting IgG antibodies specific for SARS-CoV-2 RBD and cells secreting IgG (total IgG), we performed an enzyme-linked immunospot (ELISpot) assay using the Human IgG SARS-CoV-2 RBD ELISpot^PLUS^ (HRP) kit (3850-4HPW-R1-1, Mabtech AB) according to the manufacturer’s protocol. Briefly, PBMCs were incubated for **four** days in RPMI-1640 medium with 10% fetal calf serum (FCS), supplemented with R848 (1 μg/ml; Mabtech AB) and recombinant human IL-2 (10 ng/ml) for stimulation of memory B cells. The ELISpot plates pre-coated with capturing monoclonal anti-human IgG antibodies were incubated with a total of 200,000 or 40,000 pre-stimulated cells per well for detection of RBD-specific IgG and total IgG secreting cells, respectively.

T cell responses were measured by ELISpot assay using Human IFN-γ SARS-CoV-2 ELISpot^PLUS^ kit (ALP) according to the manufacture’s protocol (3420-4AST-P1-1, Mabtech AB). Briefly, plates were washed with filtered PBS (Sigma Aldrich, Missouri, US) and blocked with RPMI-1640 culture media containing 10% batch tested fetal bovine serum (FBS) (Gibco, Thermo Fisher Scientific, Massachusetts, US). The plates pre-coated with capturing monoclonal anti-IFN-γ were incubated 18hrs in RPMI-1640 medium containing 10% FCS supplemented with a mixture containing the SARS-CoV-2 defined peptide pool, which contains 47 synthetic peptides binding to human HLA, derived from the spike protein (S), nucleoprotein (N), membrane protein (M), and the open reading frame (ORF)-3a and ORF-7a proteins (3622-1, Mabtech AB) at a concentration of 2 ug/ml of each peptide, anti-CD28 (0.1 μg/ml) and 250,000 cells per well in a humidified incubator (5% CO_2_, 37°C). Negative controls comprising DMSO and positive controls containing anti-CD3 were also included.

Spot numbers were analyzed by the CTL ImmunoSpot **S6** universal analyzer (Cellular Technology Ltd., USA). Mean values of duplicate cell cultures were considered. SARS-CoV-2-specific spots were determined as mean spots of the control wells were subtracted from the positive wells, and the results were expressed as spot-forming cells (SFC) per10^6^ PBMCs. We defined threefold higher SARS-CoV-2-specific spots versus background together with at least three spots above background as a positive response. This cutoff was set based on negative control values as described previously. If negative control wells had >30 SFC per 10^6^ PBMCs or positive control wells (anti-CD3 and CD28 stimulation) were negative, the results were excluded from further analysis.

**Immunophenotyping by flow cytometry**

The frozen PBMCs were thawed at 37°C and were washed once with the medium containing 10% FBS and RPMI-1640 (Gibco). Recovered cell viability was measured above >90% for all samples. Approximately 1×10^6^/ml were stained with live/dead mix, washed with FASC washing buffer (2% FBS, 0.1% NaN3 in PBS), and blocked with Fc receptor blocking solution. PBMCs were then incubated with fluorescence-conjugated antibodies against cell surface molecules for 25 min at 4°C. After washing with FACS buffer, the cells were fixed (eBioscience). The surface antibodies were diluted in FACS buffer with 50% BD Brilliant Buffer (BD). Samples were acquired using Cytek Aurora Spectral Flow Cytometry (Cytek Biosciences). Daily QC was performed using SpectroFlo QC beads (Cytek Biosciences) before acquiring samples. The SpectroFlo (v2.2.0 Cytek Biosciences) was used for data acquisition and analysis. Unstained controls were used for assessing autofluorescence. Single-stained cells or single-stained CompBeads (BD Biosciences) were used as reference controls for used for compensation. The antibody panel and buffer information are provided in **Table S2**. The markers used to identify each subset of cells are summarized in **Table S3**, and detailed gating strategies for the cell populations presented in the main figures are shown in **Figure S6**.

**Quantification and statistical analysis**

Categorical and continuous variables were analyzed using the χ^2^ test, Fisher's exact test, or Student's *t*-test where appropriate. One-way analysis of variance (ANOVA) (normal distribution) or Kruskal-Wallis test (failing normality testing) followed by Dunn’s multiple comparison test was used for multiple group comparisons. Multiple testing correction was subsequently applied to each comparison using the Benjamini-Hochberg (BH) procedure with a false discovery rate (FDR) at 5%. Mann-Whitney *U* test was used to compare the difference between the two groups. Spearman correlations analyses were used to determine associations between analyzed parameters. All statistical analyses were performed using GraphPad Prism (version 8.4.2 GraphPad Software, La Jolla California USA) and R software (URL <http://www.R-project.org/>). All statistical tests were 2-sided with a significance level of 0.05.

**Supplementary Table 1.** **Demographic and clinical characteristics of the COVID-19 cases and healthy controls enrolled in this study.**

| **Characteristics** | **Prolonged cases** | **Non- Prolonged cases** | **Symptomatic cases** | **Healthy controls** |
| --- | --- | --- | --- | --- |
| **No. of participants** | 6 | 24 | 20 | 20 |
| **Viral RNA shedding (days; median, IQR)** | 106.5 (100.5-109.8) | 7.0 (3.3-22.0) | NA | NA |
| **Age (median, IQR)** | 28.0 (25.8-30.0) | 30.5 (26.3-33.0) | 30.5 (29.0-32.8) | 33.5 (30.0-42.0) |
| **Sex** |  |  |  |  |
| Male | 6 (100.0) | 24 (100.0) | 20 (100.0) | 9 (45.0) |
| Female | 0 | 0 | 0 | 11 (55.0) |
| **Underlying medical condition** |  |  |  |  |
| Yes | 0 | 0 | 0 | No |
| No | 6 (100.0) | 24 (100.0) | 20 (100.0) | No |
| **Clinical symptom** |  |  |  |  |
| Yes | 0 | 0 | 20 (100.0) | No |
| No | 6 (100.0) | 24 (100.0) | 0 | No |

**IQR, interquartile range.**

**Supplementary Table 2. Comparison of variations within the S and ORF1a/b genes.**

| **Variations** | **Genes** | | ***p* value** |
| --- | --- | --- | --- |
|  | **S** | **ORF1a** |  |
| All amino acids | 1273 | 7096 | 0.00003 |
| Nonsynonymous variations | 33 | 78 |  |
|  |  |  |  |
| All amino acids in S | 1273 | 7096 | 0.63 |
| Synonymous SNVs | 10 | 47 |  |
|  |  |  |  |
| All amino acids in S | 1273 | 4405 | 0.003 |
| Nonsynonymous SNVs | 33 | 60 |  |
|  |  |  |  |
| All amino acids in S | 1273 | 4405 | 0.036 |
| Synonymous SNVs | 10 | 15 |  |

OFR, open reading frame; S, spike; SNV, single nucleotide variation. Two-sided Chi-square test was used for the comparison of enrichments of non-synonymous and synonymous variations within the S gene, compared with the ORF1a and ORF1b genes.

**Supplementary Table 3. Reagents used in flow cytometry**

| **Reagent** | **Clone** | **Dilution** | **Source** | **Identifier** |
| --- | --- | --- | --- | --- |
| **Antibodies** |  |  |  |  |
| Brilliant Violet 785™ anti-human CD14 | M5E2 | 400 | Biolegend | 301840 |
| Brilliant Violet 650™ anti-human CD16 | 3G8 | 400 | Biolegend | 302042 |
| PerCP/Cyanine5.5 anti-human CD3 | SK7 | 400 | Biolegend | 344808 |
| PE anti-human CD19 | SJ25C1 | 400 | Biolegend | 363004 |
| PE/Cyanine7 anti-human CD27 | M-T271 | 200 | Biolegend | 356412 |
| APC/Cyanine7 anti-human CD38 | HB-7 | 400 | Biolegend | 356616 |
| Pacific Blue™ anti-human CD8 | SK1 | 400 | Biolegend | 344718 |
| Brilliant Violet 750™ anti-human CD56 | 5.1H11 | 400 | Biolegend | 362556 |
| PE/Dazzle™ 594 anti-human CD57 | HNK-1 | 400 | Biolegend | 359620 |
| Brilliant Violet 510™ anti-human CD4 | SK3 | 400 | Biolegend | 344634 |
| Brilliant Violet 711™ anti-human CD45RA | HI100 | 800 | Biolegend | 304138 |
| FITC anti-human HLA-DR | L243 | 400 | Biolegend | 307604 |
| APC anti-human CD45RO | UCHL1 | 500 | Biolegend | 304210 |
| Brilliant Violet 605™ anti-human CD127 | A019D5 | 200 | Biolegend | 351334 |
| Brilliant Violet 421™ anti-human CD25 | BC96 | 200 | Biolegend | 302630 |
| **Buffer** |  |  |  |  |
| BD Horizon™ Brilliant Stain Buffer | NA | NA | BD Bioscienes | 566349 |
| BD Pharmingen™ Transcription  Factor Buffer Set | NA | NA | BD Bioscienes | 562574 |
| **QC beads** |  |  |  |  |
| Spectro-Flo QC Beads | NA | NA | Cytek | SKU N7-97355 |
| **Compbead** |  |  |  |  |
| BD™ CompBead Anti-Mouse Ig, κ/Negative Control Compensation Particles Set | NA | NA | BD Bioscienes | 552843 |
| BD™ CompBead Anti-Rat and Anti-Hamster Ig κ /Negative Control Compensation Particles Set | NA | NA | BD Bioscienes | 552845 |
| **Live/Dead buffer** |  |  |  |  |
| 0.1% Zombie NIR™ Fixable Viability | NA | NA | BioLegend | 423105 |
| 99.9% 1x DPBS | NA | NA | Corning | 21-031-CV |
| **10% RPMI** |  |  |  |  |
| RPMI-1640 | NA | NA | Gibco | C11875500BT |
| 10% Fetal Bovine Serum | NA | NA | Gibco | 10270-106 |
| 1% Penicillin-Streptomycin | NA | NA | Gibco | 15140122 |
| **Fc block** |  |  |  |  |
| 90% FACS buffer | NA | NA |  |  |
| 10% Human Fc block | NA | NA | BioLegend | 422302 |
| **FACS buffer** |  |  |  |  |
| 1x DPBS | NA | NA | Corning | 21-031-CV |
| 1% Fetal Bovine Serum | NA | NA | Gibco | 10270-106 |
| **Perm/Wash buffer** |  |  |  |  |
| 20% TF Perm/Wash Buffer | NA | NA | BD Bioscienes | 562574 |
| 80% DI Water |  |  |  |  |
| **Fix/Perm buffer** |  |  |  |  |
| 25% TF Fix/Perm | NA | NA | BD Bioscienes | 562574 |
| 75% TF Diluent Buffer | NA | NA | BD Bioscienes | 562574 |

**Supplementary Table 4. Definitions of each cell subset in flow cytometry with specific antibody markers.**

| **Flow cytometry staining** | **Cell population** | **Definition by markers** |
| --- | --- | --- |
| PBMC cell composition staining (by surface markers) |  |  |
|  | B | CD19+CD3- |
|  | T | CD19-CD3+ |
|  | Plasma | CD19+CD3-/CD38+CD27- |
|  | Plasmablast | CD19+CD3-/CD38+CD27+ |
|  | Memory B | CD19+CD3-/CD38-CD27+ |
|  | CD56++CD16- NK | CD19-CD3-/CD56++CD16- |
|  | CD56+CD16+ NK | CD19-CD3-/CD56+CD16+ |
|  | Classical monocytes | CD19-CD3-/CD14+CD16- |
|  | Intermediate monocytes | CD19-CD3-/CD14+CD16+ |
| T cells subsets by surface makers |  |  |
|  | CD4 T | CD19-CD3+/CD8-CD4+ |
|  | Treg | CD19-CD3+/CD8-CD4+/CD127dimCD25hiFoxp3+ |
|  | Naive CD4 T | CD19-CD3+/CD8-CD4+/CD45RO-CD45RA+ |
|  | Memory CD4 T (CD4 T_M_) | CD19-CD3+/CD8-CD4+/CD45RO+CD45RA- |
|  | Effect CD4 T (CD4 T_E_) | CD19-CD3+/CD8-CD4+/CD57+CD45RA- |
|  | Terminal effect CD4 T (CD4 T_TE_) | CD19-CD3+/CD8-CD4+/CD57+CD45RA+ |
|  | Effect memory CD4 T (CD4 T_EM_) | CD19-CD3+/CD8-CD4+/CD45RO+CD45RA-/CD27- |
|  | Central memory CD4 T (CD4 T_CM_) | CD19-CD3+/CD8-CD4+/CD45RO+CD45RA-/CD27+ |
|  | TCR-activated CD4 T | CD19-CD3+/CD8-CD4+/HLA-DR+CD38+ |
|  | CD8 T | CD19-CD3+/CD8+CD4- |
|  | Naive CD8 T | CD19-CD3+/CD8+CD4-/CD45RO-CD45RA+ |
|  | Memory CD8 T (CD8 T_M_) | CD19-CD3+/CD8+CD4-/CD45RO+CD45RA- |
|  | Effect CD8 T (CD8 T_E_) | CD19-CD3+/CD8+CD4-/CD57+CD45RA- |
|  | Terminal effect CD8 T (CD8 T_TE_) | CD19-CD3+/CD8+CD4-/CD57+CD45RA+ |
|  | Effect memory CD8 T (CD8 T_EM_) | CD19-CD3+/CD8+CD4-/CD45RO+CD45RA-/CD27- |
|  | Central memory CD8 T (CD8 T_CM_) | CD19-CD3+/CD8+CD4-/CD45RO+CD45RA-/CD27+ |
|  | TCR-activated CD8 T | CD19-CD3+/CD8+CD4-/HLA-DR+CD38+ |

**Supplementary Figures**

**Supplementary Fig. 1. Phylogenetic lineages of the SARS-CoV-2 genomes.** **a**. The maximum likelihood unrooted phylogenetic tree of SARS-CoV-2 genomes (n = 69) in this study and approximately 1000 public SARS-CoV-2 genomes public genomes from the GISAID database (till December 17, 2020) from six continents and representatives of the global viral lineages. The phylogenetic tree was constructed with 1000 bootstraps, and the scale represents 0.0001 nucleotide substitutions per site. The genomes from patients in this study were highlighted with a gray shadow, and the dominating lineages of the viral genomes in the two clades were labeled aside (lineages B.1 and B.4). **b-f** The phylogenetic analysis of genomes from sequential samples. In each of the five cases (Cases 1 to 5), a phylogenetic tree of the obtained genomes and 210 representative public genomes indicated in grey color were constructed, as in **a**. The blue and red tree branches denote the lineages B.1 and B.4 genomes, respectively.


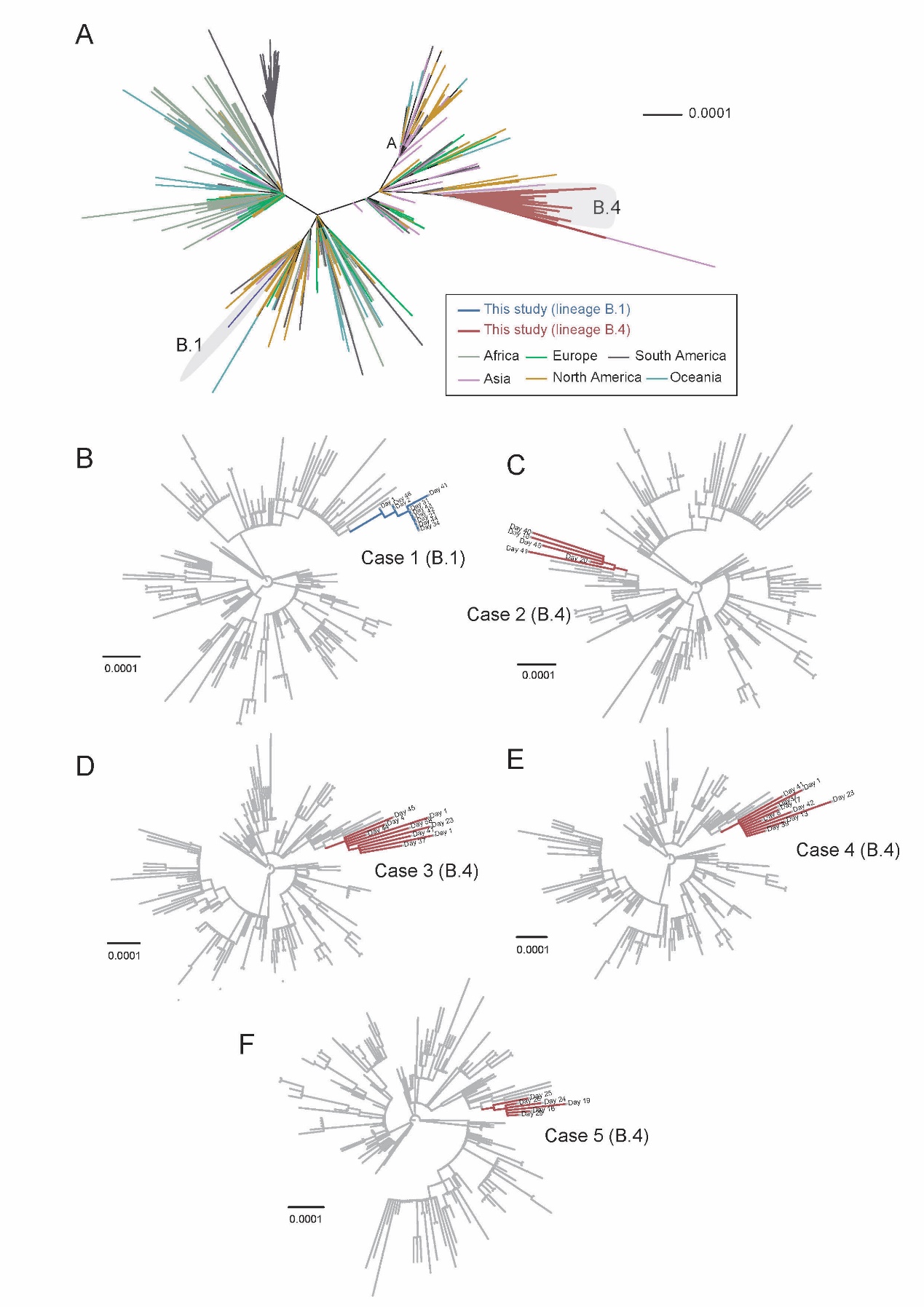


**Supplementary Fig. 2. Genomic variation of the SARS-CoV-2 genomes.** **a**. The maximum likelihood phylogenetic tree of the 69 genomes, with Wuhan-hu-1 strain as a reference genome (GenBank accession MN908947.3, GISAID accession EPI_ISL_402125). The genomes from the persistently infected individuals (C1 – C6, n = 40) are denoted in chromatic colors, while non-persistent cases were in grey (n = 29). **b.** Consensus-level substitutions of the 69 SARS-CoV-2 genomes. The genomes were aligned with the corresponding branches of the phylogenetic tree in A. Substitutions are denoted with solid dots. The characteristic loci for lineage B.1 and lineage B.4 are denoted at the top in blue and red, respectively. NC, non-coding. Nonsyn, nonsynonymous. Syn, synonymous. Stop, stop gain. NA, not available, which indicates the substitutions had insufficient supporting sequencing reads. **c**. The B.1 viruses had significant higher titers than the B.4 viruses. The cycle threshold (C_t_) values for SARS-COV2-open reading frame 1ab (OFR1ab) of RT-PCR are exhibited at box-plots, with the *p*-value of two-sided Wilcoxon rank-sun test. Boxplots indicate median, IQR, and 95% confidence interval.


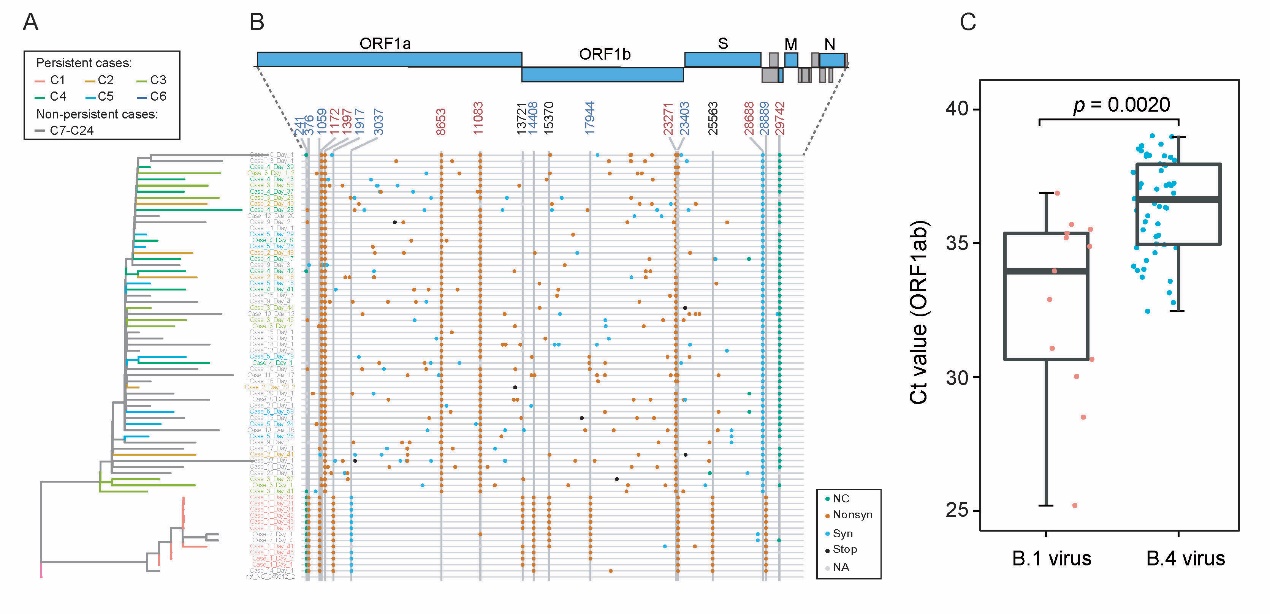


**Supplementary Fig. 3.** **IgG antibody to the spike and RBD antigens of SARS-CoV2 and neutralizing antibody to pseudovirus SARS-CoV-2 in healthy controls**. Analysis of anti-spike IgG, anti-RBD IgG, and neutralizing antibody (NAb) levels in serum from 20 pre-COVID-19 negative healthy controls. The geometric mean (endpoint) titers (red line) and 95%CI are shown for the IgG and NAb at timepoints of serum sampling.


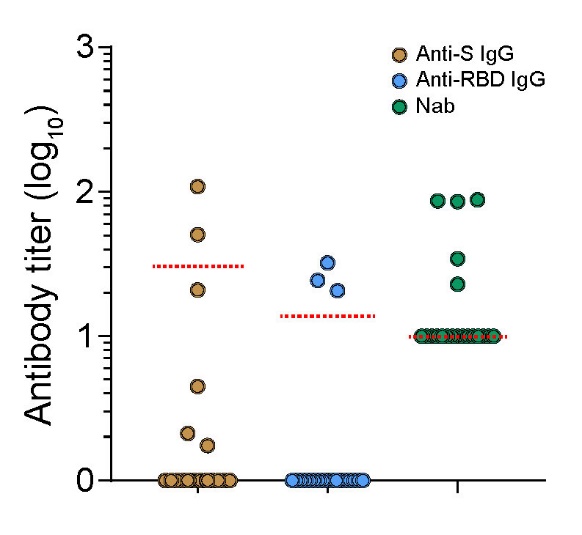


**Supplementary Fig. 4. Correlations between SARS-CoV-2-specific NAb titer and anti-spike (S), and -receptor-binding domain (RBD) IgG levels, and the correlation between anti-S IgG and anti-S IgG.** Statistical comparisons were performed using the two-sided nonparametric Spearman correlation.


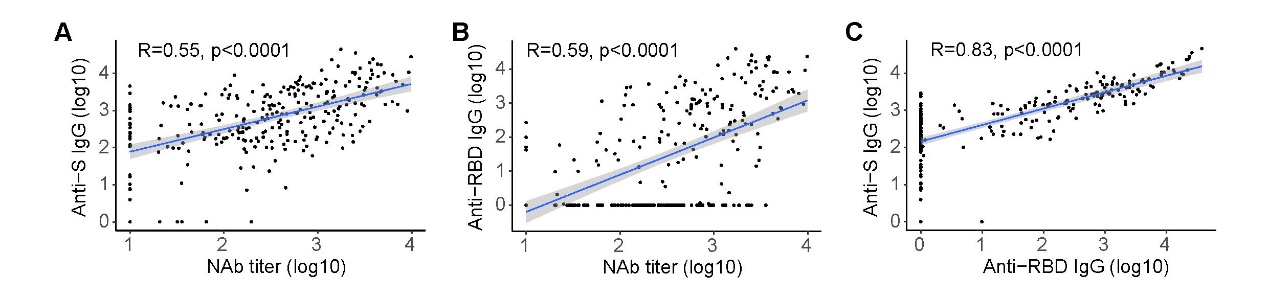


**Supplementary Fig. 5.** **Correlations between antibody response and specific B and T cell response.** Statistical comparisons were performed using the two-sided nonparametric Spearman correlation.


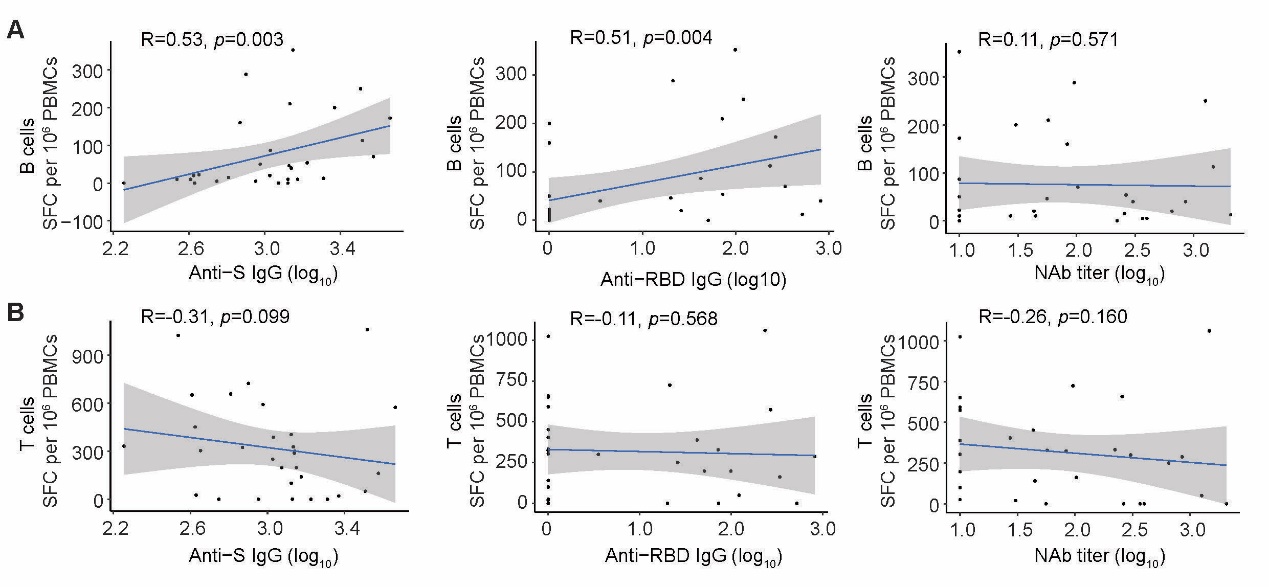


**Supplementary Fig. 6.** **Flow cytometry gating strategy for immune cell subsets, Related to Fig. 1d.** The proportion of B cells in live PBMCs and frequency of plasma cells and plasmablast in B cells, the frequency of monocytes subsets in monocytes and NK subsets in NK cells, the proportion of T cells in live PBMCs and frequency of CD4 and CD8 T cells in T cells, the frequency of activated CD4 T cells and maturation of CD4 T cells in CD4 T cells and the frequency of activated CD8 T cells and maturation of CD8 T cells in CD8 T cells were presented in Fig. 1d.


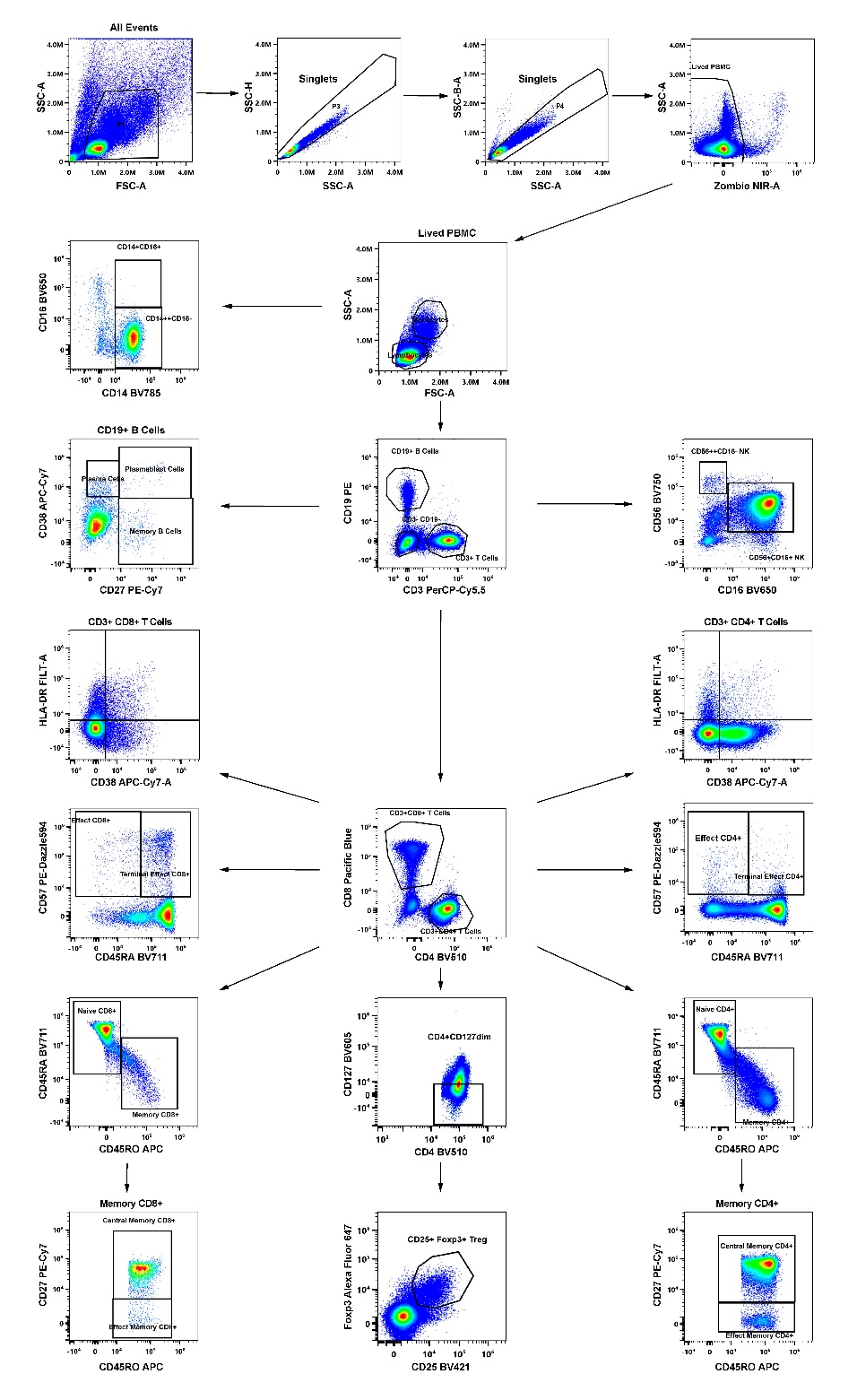


**References**

1 Wolfel, R. *et al.* Virological assessment of hospitalized patients with COVID-2019. *Nature* **581**, 465-469, doi:10.1038/s41586-020-2196-x (2020).

2 Chen, S., Zhou, Y., Chen, Y. & Gu, J. fastp: an ultra-fast all-in-one FASTQ preprocessor. *Bioinformatics (Oxford, England)* **34**, i884-i890, doi:10.1093/bioinformatics/bty560 (2018).

3 Bolger, A. M., Lohse, M. & Usadel, B. Trimmomatic: a flexible trimmer for Illumina sequence data. *Bioinformatics (Oxford, England)* **30**, 2114-2120, doi:10.1093/bioinformatics/btu170 (2014).

4 Li, H. & Durbin, R. Fast and accurate short read alignment with Burrows-Wheeler transform. *Bioinformatics (Oxford, England)* **25**, 1754-1760, doi:10.1093/bioinformatics/btp324 (2009).

5 Xiao, K. *et al.* Isolation of SARS-CoV-2-related coronavirus from Malayan pangolins. *Nature*, doi:10.1038/s41586-020-2313-x (2020).

6 Li, H. *et al.* The Sequence Alignment/Map format and SAMtools. *Bioinformatics (Oxford, England)* **25**, 2078-2079, doi:10.1093/bioinformatics/btp352 (2009).

7 Koboldt, D. C. *et al.* VarScan 2: somatic mutation and copy number alteration discovery in cancer by exome sequencing. *Genome Res* **22**, 568-576, doi:10.1101/gr.129684.111 (2012).

8 Ye, K., Schulz, M. H., Long, Q., Apweiler, R. & Ning, Z. Pindel: a pattern growth approach to detect break points of large deletions and medium sized insertions from paired-end short reads. *Bioinformatics (Oxford, England)* **25**, 2865-2871, doi:10.1093/bioinformatics/btp394 (2009).

9 Thorvaldsdóttir, H., Robinson, J. T. & Mesirov, J. P. Integrative Genomics Viewer (IGV): high-performance genomics data visualization and exploration. *Brief Bioinform* **14**, 178-192, doi:10.1093/bib/bbs017 (2013).

10 Cingolani, P. *et al.* A program for annotating and predicting the effects of single nucleotide polymorphisms, SnpEff: SNPs in the genome of Drosophila melanogaster strain w1118; iso-2; iso-3. *Fly (Austin)* **6**, 80-92, doi:10.4161/fly.19695 (2012).

11 Rambaut, A. *et al.* A dynamic nomenclature proposal for SARS-CoV-2 lineages to assist genomic epidemiology. *Nature microbiology* **5**, 1403-1407, doi:10.1038/s41564-020-0770-5 (2020).

12 Katoh, K., Misawa, K., Kuma, K. & Miyata, T. MAFFT: a novel method for rapid multiple sequence alignment based on fast Fourier transform. *Nucleic acids research* **30**, 3059-3066, doi:10.1093/nar/gkf436 (2002).

13 Kumar, S., Stecher, G., Li, M., Knyaz, C. & Tamura, K. MEGA X: Molecular Evolutionary Genetics Analysis across Computing Platforms. *Molecular biology and evolution* **35**, 1547-1549, doi:10.1093/molbev/msy096 (2018).

14 Minh, B. Q. *et al.* IQ-TREE 2: New Models and Efficient Methods for Phylogenetic Inference in the Genomic Era. *Molecular biology and evolution* **37**, 1530-1534, doi:10.1093/molbev/msaa015 (2020).

15 Stamatakis, A. RAxML version 8: a tool for phylogenetic analysis and post-analysis of large phylogenies. *Bioinformatics (Oxford, England)* **30**, 1312-1313, doi:10.1093/bioinformatics/btu033 (2014).

16 Letunic, I. & Bork, P. Interactive Tree Of Life (iTOL): an online tool for phylogenetic tree display and annotation. *Bioinformatics (Oxford, England)* **23**, 127-128, doi:10.1093/bioinformatics/btl529 (2007).

17 Choi, B. *et al.* Persistence and Evolution of SARS-CoV-2 in an Immunocompromised Host. *N Engl J Med* **383**, 2291-2293, doi:10.1056/NEJMc2031364 (2020).

18 Ju, B. *et al.* Human neutralizing antibodies elicited by SARS-CoV-2 infection. *Nature* **584**, 115-119, doi:10.1038/s41586-020-2380-z (2020).
